# A continental-scale soil evaporation dataset derived from Soil Moisture Active Passive satellite drying rates

**DOI:** 10.1038/s41597-020-00748-z

**Published:** 2020-11-20

**Authors:** Ronnie Abolafia-Rosenzweig, Andrew M. Badger, Eric E. Small, Ben Livneh

**Affiliations:** 1 Department of Civil, Environmental, and Architectural Engineering, University of Colorado Boulder, Boulder, CO 80309 USA; 2 Universities Space Research Association, Columbia, MD 21046 USA; 3 Hydrological Sciences Laboratory, NASA Goddard Space Flight Center, Greenbelt, MD 20771 USA; 4 Geological Sciences, University of Colorado Boulder, Boulder, CO 80309 USA; 5 Cooperative Institute for Research in Environmental Sciences (CIRES), University of Colorado Boulder, Boulder, CO 80309 USA

**Keywords:** Civil engineering, Hydrology, Hydrology

## Abstract

This manuscript describes an observationally-based dataset of soil evaporation for the conterminous U.S. (CONUS), gridded to a 9 km resolution for the time-period of April 2015-March 2019. This product is termed E-SMAP (Evaporation-Soil Moisture Active Passive) in which soil evaporation is estimated from the surface layer, defined by the SMAP sensing depth of 50 mm, between SMAP overpass intervals that are screened on the basis of precipitation and SMAP quality control flags. Soil evaporation is estimated using a water balance of the surface soil that we show is largely dominated by SMAP-observed soil drying. E-SMAP soil evaporation is on average 0.72 mm day^−1^, which falls within the range of soil evaporation estimates (0.17–0.89 mm day^−1^) derived from operational land surface models and an alternative remote sensing product. E-SMAP is independent from existing soil evaporation estimates and therefore has the potential to improve understanding of evapotranspiration partitioning and model development.

## Abstract (summary)

**Table:** 

Measurement(s)	evaporation
Technology Type(s)	satellite imaging • computational modeling technique
Factor Type(s)	geographic location • temporal interval
Sample Characteristic - Environment	soil
Sample Characteristic - Location	United States of America

Machine-accessible metadata file describing the reported data: 10.6084/m9.figshare.13181822

## Background & Summary

Evapotranspiration (*ET*) connects the surface water and energy budgets^1^. It is the second largest component of the terrestrial water balance after precipitation and is a source of feedback in the climate system^2,3^. Our ability to observe the return-flow of moisture from the land to the atmosphere is limited by sparse *in situ* observations that are not generally representative of regional scales^4–6^. Remotely sensed *ET* across a range of data products often have similar representations of *ET*’s seasonality^2,7^. However, these products show large dissimilarities^7^, in particular when water is the limiting factor for *ET* (e.g. during drought)^2,8^. In the absence of snow, *ET* is the sum of three components: 1) evaporation from the soil surface (*E*_*soil*_), 2) transpiration from vegetation (*E*_*T*_), and 3) evaporation of intercepted water from vegetation canopies (*E*_*C*_). Partitioning of *ET* into these three components with models^5,9,10^ and remote sensing^2^ often reveal large disagreements. In this study, we apply the methodology developed by Small *et al*.^11^ to estimate soil evaporation using soil moisture drying rates observed by the Soil Moisture Active Passive (SMAP) satellite. This continental-scale gridded dataset is unique from other datasets and has the potential to improve the representation of *ET* partitioning in hydrologic models and climate studies.

Ground-based observational techniques, for example, the eddy covariance^12^ or Bowen Ratio energy balance methods^13,14^, provide measurements of the total *ET* flux. However, these ground-based observations only provide estimates of *E*_*soil*_ when *E*_*T*_ is zero, for example when vegetation experiences seasonal senescence. Ground-based measurements can provide estimates of *E*_*soil*_ directly, such as weighing lysimeters^15,16^, and indirectly, such as the heat pulse method^17,18^. However, such ground-based observations of *E*_*soil*_ are labor intensive, and thus cannot be applied at the regional scale or for long-term monitoring^15–19^.

Land surface models (LSMs) compliment sparse ground-based monitoring of *ET* by producing spatially and temporally continuous estimates of total *ET* and its components. Yet, simulated fluxes are dependent on imperfect model structure and parameters that are difficult to estimate, resulting in large differences in *E*_*soil*_ estimates from different LSMs^4,5,11^. Total *ET* simulated by LSMs in the Global Land Data Assimilation System (GLDAS^20^), North American Land Data Assimilation System phase 2 (NLDAS-2^21,22^) and experimental NLDAS-Testbed have been evaluated through comparison with remotely sensed *ET*^23,24^ and networks of eddy covariance flux towers^11,25^, but there has been no similar effort to evaluate *E*_*soil*_, *E*_*T*_ or *E*_*c*_ as few datasets exist for this purpose^4,5^. Without observationally-based estimates of how *ET* is partitioned into the component fluxes, it is not possible to improve the representation of *E*_*soil*_, *E*_*T*_ or *E*_*c*_ in hydrologic models.

Remote sensing provides a promising tool for estimating latent heat flux and evaluating simulated *ET*. Remote sensing methods that estimate *ET* largely rely on thermal data as a key input to the evapotranspiration algorithm^26–29^. However, these algorithms do not provide information about *ET* partitioning, only the total *ET* flux. Two exceptions being the Global Land Evaporation Amsterdam Model (GLEAM^3,30^) and the Priestly Taylor Jet Propulsion Laboratory (PT-JPL^31,32^) products, that provide estimates of total *ET* and its components. These realizations use remotely sensed soil moisture to inform estimates of *E*_*soil*_, but both GLEAM and PT-JPL are strongly dependent on models that indirectly estimate *ET* and its components rather than direct measurements of evaporative flux (e.g. weighing lysimeters).

**Fig. 1:**
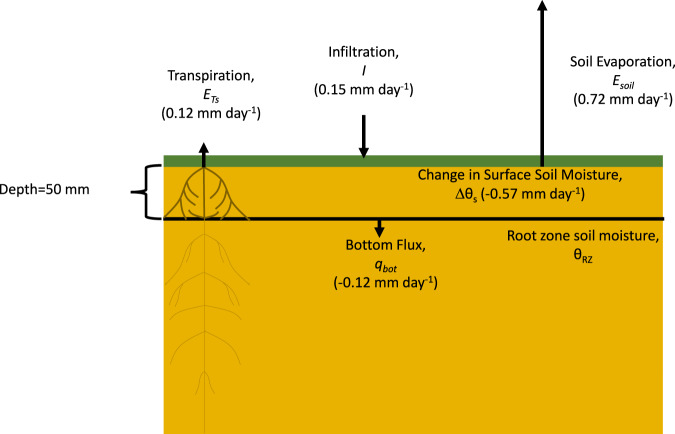
The water balance framework used to estimate soil evaporation. The E-SMAP approach is analogous to using SMAP as a lysimeter with a sensing scale equivalent to SMAP’s 9 km × 9 km footprint. Soil evaporation, *E*_*soil*_, is estimated by Eq. 1 that accounts for fluxes in and out of a control volume (50 mm surface soil layer observed by SMAP). The direction of arrows represents the sign convention in Eq. 1 and the size of the arrow is proportional to the mean magnitude of each flux over intervals with minimal precipitation where E-SMAP records *E*_*soil*_. The transpiration flux, *E*_*Ts*_, only includes water extracted by roots in the surface layer.

To address the above issues, we develop a new remote sensing-based dataset of *E*_*soil*_ over the conterminous United States (CONUS) from 2015–2019 that essentially uses SMAP as a giant lysimeter with a sensing scale equivalent to SMAP’s 9 km x 9 km footprint. This Evaporation-Soil Moisture Active Passive dataset (E-SMAP) is the first to use remotely sensed soil drying rates in a mass balance framework to estimate *E*_*soil*_ (Fig. 1), thus providing unique estimates of *E*_*soil*_^3,31^. We extend the initial work of Small *et al*.^11^ that developed and evaluated E-SMAP at several *in situ* observation locations, to provide a continental-scale, 4-year, 9 km soil evaporation dataset. In this data descriptor, we first describe calculation of *E*_*soil*_ and a data screening procedure, followed by an exposition into the components of soil evaporation. Since there are no ‘true’ observations of continental-scale soil evaporation, the technical evaluation consists of comparisons between E-SMAP and another remote sensing *E*_*soil*_ product (GLEAM) as well as two LSM-based datasets from the NLDAS-2.

## Methods

### Evaporation and the water balance of the surface soil layer

The procedure used to create E-SMAP follows the methodology described in Small *et al*.^11^. A brief summary is provided here, along with descriptions of alterations made to that approach. *E*_*soil*_ is estimated independently at each SMAP 9 km x 9 km grid cell via a water balance of the surface soil control volume (Fig. 1), where:

1 $${E}_{soil}=-\frac{d{\theta }_{s}}{dt}D-{q}_{bot}-{E}_{Ts}+I$$

${\theta }_{s}$ is volumetric soil moisture in the surface soil control volume (mm^3^mm^−3^), *D* is the thickness of the control volume (mm)*, q*_*bot*_ (mm day^−1^) is the flux across the bottom boundary of the control volume, *E*_*Ts*_ (mm day^−1^) is surface transpiration which is the fraction of total transpiration proportional to the fraction of roots within the top 50 mm surface soil layer, and *I* is infiltration (mm day^−1^). We define the thickness of the control volume, *D*, to be equivalent to the SMAP sensing depth (50 mm)^33^, noting that this sensing depth can vary through time with soil moisture^34^. We define *q*_*bot*_ as positive when water moves from the control volume to deeper soil and negative when water moves from deeper soil to the control volume. Surface transpiration, *E*_*Ts*_, is the fraction of total *E*_*T*_, proportional to the fraction of roots within the top 50 mm of the soil.

We use SMAP soil moisture time series to estimate *E*_*soil*_ following the assumption that *E*_*soil*_ is typically the largest flux in Eq. 1 excluding times when infiltration is actively occurring due to precipitation or snowmelt^11^. The observed ${\theta }_{s}$ time series is used to calculate $\frac{d{\theta }_{s}}{dt}$ for intervals defined by successive SMAP overpasses^35^. The remaining terms on the right-hand side of Eq. 1 are estimated using a combination of auxiliary data and models described below.

### Precipitation screening

**Fig. 2:**
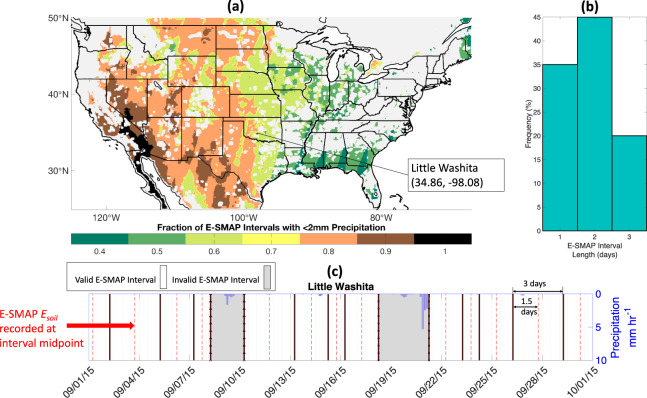
Valid E-SMAP intervals and screening procedure. **(a)** E-SMAP’s spatial domain is shaded by the fraction of valid E-SMAP intervals on the basis of minimal precipitation (less than 2 mm). Land surface areas screened based on SMAP’s quality flag and Hydrus non-convergence are masked in white. **(b)** a histogram of valid E-SMAP interval durations. **(c)** an example time series to illustrate E-SMAP’s recording and screening procedure during one month over the Little Washita location. SMAP observations (black lines) are all at 6 AM local time. E-SMAP soil evaporation is coded at the midpoint of each E-SMAP interval (red dashed lines). E-SMAP intervals with more than 2 mm of cumulative precipitation are screened (grey shading). Drying rates are calculated from successive 6 AM SMAP observations bounding the E-SMAP interval, and other fluxes on the right-hand-side of Eq. 1 are estimated at finer time steps (hourly) and summed over the E-SMAP interval.

Following Small *et al*.^11^, Eq. 1 is not applied to SMAP overpass intervals with substantial precipitation, since we seek to minimize uncertainties in the partitioning of incoming precipitation between runoff, canopy interception, and infiltration. Therefore, ‘valid intervals’ are defined as successive SMAP overpasses with less than 2 mm of precipitation, while those with larger precipitation values are considered ‘not valid’^11^. This threshold was selected to reflect SMAP’s accuracy and sensing depth^33,36^, where 2 mm of infiltrated water in a 50 mm soil column yields a soil moisture change equal to SMAP’s reported uncertainty (0.04 mm^3^mm^−3^). After screening for precipitation, 66% of SMAP’s overpasses remain valid (Fig. 2a).

### Bottom flux (q_bot_)

We use the Hydrus 1-D model^37^ to estimate *q*_*bot*_. Model inputs include soil properties that are defined using soil texture and top boundary conditions that are set to observed atmospheric boundary conditions (Table 1). The model solves the Richards’ equation for saturated and unsaturated conditions. Here, the modeled soil column depth was set to 1000 mm, discretized with 101 nodes evenly separated 10 mm apart. Model simulations were initialized with a 4 year run (April 1, 2015- March 31, 2019), where the outputs from March 31, 2019 of this spin-up were used to set initial soil moisture conditions in the Hydrus simulations used to calculate *q*_*bot*_ for E-SMAP. The exchange of moisture below the 50 mm node represents the flux at the bottom boundary of the control volume, *q*_*bot*_. Small *et al*.^11^ quantified the uncertainty of *q*_*bot*_ caused by soil parameter uncertainties to be less than 0.1 mm day^−1^ during valid intervals (<2 mm of total precipitation).

**Table 1:** Data sources used to build the E-SMAP dataset.

Data type	Source	Citation
Soil moisture	SMAP Enhanced L3 radiometer Global Daily 9 km EASE-Grid Soil Moisture, Version 1 (https://nsidc.org/data/smap/smap-data.html)	O’Neill *et al*.^48^
Meteorological forcing (precipitation, surface pressure, temperature, specific humidity)	NLDAS-2 (https://hydro1.gesdisc.eosdis.nasa.gov/data/NLDAS/NLDAS_FORB0125_H.002/)	Xia *et al*.^21^; Xia *et al*.^22^; NCEP/EMC^49^
Enhanced vegetation index	MOD13 A2 (https://e4ftl01.cr.usgs.gov/MOLT/)	Didan *et al*.^40^
Vegetation classification	UMD Land Cover Classification from AVHRR (https://data.mint.isi.edu/files/raw-data/land-use/USGS_LCI/GLCF%3A%20AVHRR%20Global%20Land%20Cover%20Classification.pdf)	Hansen *et al*.^50^; Hansen *et al*.^51^
Soil properties	Texture type: NLDAS-2 (Miller and White, 1998; https://ldas.gsfc.nasa.gov/nldas/soils) Parameters: NCAR (https://ral.ucar.edu/sites/default/files/public/product-tool/noah-multiparameterization-land-surface-model-noah-mp-lsm/SOILPARM.TBL_.txt) Van Genuchten parameters: USDA (https://www.ars.usda.gov/ARSUserFiles/80420525/EnvironmentalTransport/CalcPTFFiles/PTF_Manual.version_3.0.pdf) Saturated Hydraulic Conductivity (Chen and Dudhia, 2001).	Miller and White^52^; Chen and Dudhia^53^

### Transpiration from the surface soil layer (E_Ts_)

We compute transpiration from the surface soil control volume for each grid cell based based on the calculation of total transpiration^38^. Using a modified version of the Penman-Monteith potential evapotranspiration (PET) equation^39^, potential transpiration is calculated accounting for fraction of the land surface covered by vegetation based on Enhanced Vegetation Index (EVI)^40^:

2 $$\lambda E=\frac{\left(s\times A\times {F}_{c}+\rho \times {C}_{p}\times \frac{{e}_{sat}-e}{{r}_{a}}\right)\times (1-{F}_{wet})}{s+\gamma \times \left(1+\frac{{r}_{s}}{{r}_{a}}\right)}$$

where *λE* is potential transpiration, *s* is the slope of the saturated water vapor pressure curve (Pa K^−1^), *A* is the net radiation (W m^−2^), $\rho$ is air density (kg m^−3^), *C*_*p*_ is specific heat capacity of air (1005 J kg^−1^ K^−1^), *e*_*sat*_-*e* is vapor pressure deficit, *r*_*a*_ is aero dynamic resistance (s m^−1^), $\gamma$ is the psychometric constant (Pa K^−1^), and *r*_*s*_ is surface resistance. *F*_*c*_ is the fraction of total vegetation cover calculated as a function of EVI^38^ and *F*_*wet*_ is the relative surface wetness^38^. We then calculate *E*_*Ts*_ from *λE* by applying linear restrictions based on the fraction of total roots in the surface soil layer following an exponential function for root density^41^ as well as the surface soil water stress using observed soil moisture content from SMAP and soil properties^42,43^ in Eq. 3

3 $${E}_{Ts}=(\lambda E\times rf)\times {F}_{SM}$$

where *rf* is the percent of roots in the top 50 mm of the surface soil column^41^ and *F*_*SM*_ is the soil water stress, calculated following prior literature^42,43^ using Eq. 4

4 $${F}_{SM}=\frac{({\theta }_{i}-{\theta }_{w})}{({\theta }_{cap}-{\theta }_{w})}$$

where *θ*_*i*_ is soil moisture at timestep *i*, *θ*_*w*_ is the wilting point of the soil and *θ*_*cap*_ is the field capacity of the soil.

Input data sources for calculation of *E*_*Ts*_ can be found in Table 1.

### Infiltration (I)

*I* is assumed to be equivalent to precipitation during valid intervals, and is therefore expected to be overestimated since canopy interception is not considered. We do not expect this error source to significantly impact *E*_*soil*_ calculated over intervals with little or no precipitation because overestimates in *I* will largely cancel out with overestimates in downwards *q*_*bot*_ which are estimated from Hydrus 1-D simulations that receive the same precipitation. This assumption may result in underestimation of *E*_*soil*_ during periods when *I* is driven by other sources, such as snowmelt. However, these errors are expected to negligibly impact E-SMAP because SMAP already includes screening flags for regions and times with frozen soil and substantial snow coverage (snow fraction exceeding 5%)^44^.

### Data screening

Data are screened on the basis of precipitation (described above in the Precipitation Screening section) as well as through SMAP quality flags. SMAP’s retrieval quality flag is used to screen data that is not of “recommended quality”^44^. Screening on the basis of SMAP’s quality flags resulted in a reduction of nearly 40% of all SMAP grid cells in the study domain (118,531 to 72,105).

An additional constraint is the non-convergence of the Hydrus 1-D solver. 9,450 grid cells did not converge in Hydrus 1-D with the originally chosen soil parameter sets. To overcome the non-convergence, soil parameters at these grid cells were altered one of two ways: (1) parameters associated with the secondary soil classification at the grid cell were used or (2) if there was not a secondary soil classification, the NLDAS-2 “other” soil classification was used. Altering soil parameters resulted in convergence of 8,699 grid cells, while the remaining 751 points (0.6% of the domain) were ultimately screened from the dataset. Altering soil parameters is expected to have minimal impacts on calculations of *E*_*soil*_ because the uncertainty in *q*_*bot*_ associated with soil parameters is much smaller than the magnitude of *E*_*soil*_^11^. Finally, intervals with negative *E*_*soil*_ or *E*_*Ts*_ estimates were considered physically unrealistic and were also screened, reducing the E-SMAP space-time domain by 31%. The two primary reasons for negative *E*_*soil*_ outputs from Eq. 1 are (i) negative biases in SMAP observed drying rates and (ii) underestimates in precipitation (e.g. under-catch errors). The implications of this screening procedure as a whole are presented in the Technical Evaluation section.

### Statistical testing

Statistical significance of a Pearson correlation reported in the Technical Evaluation section is calculated from a right-tailed significance test in MATLAB (https://www.mathworks.com/help/stats/corr.html). Statistical significance of the differences between medians that are reported in the Technical Evaluation section are calculated from paired one-tailed Wilcoxon signed rank tests using the exactRankTests R Library^45^.

## Data Records

A list of data sources used to build E-SMAP are included in Table 1. Each data source is remapped to SMAP’s 9 km EASE-Grid with the nearest neighbor approach. As part of the E-SMAP dataset, gridded estimates are posted for each component in Eq. 1 on SMAP’s 9 km EASE-Grid from April 2015 through March 2019 during SMAP’s valid intervals (Table 2). The spatial domain encompasses 25°N–50°N and 125°W–67°W, covering the entire CONUS. The dataset, archived on Mendeley in netCDF format, is intended to support modeling development efforts that focus on the partitioning of *ET* into its components and climate case studies within the period of data record (2015–2019) that require independent representation of *ET* components. The dataset should be cited as: Abolafia-Rosenzweig, R., Badger, A., Small, E., Livneh, B. E-SMAP: Evaporation-Soil Moisture Active Passive. *Mendeley* 10.17632/ffw8zbdmpm.2 (2020)^46^.

**Table 2:** List of publicly available variables included in the E-SMAP dataset.

Variable name	Units
Soil Evaporation (*E*_*soil*_)	mm day^−1^
Soil Moisture Flux $(\frac{d{\theta }_{s}}{dt}D)$	mm day^−1^
Bottom flux (*q*_*bot*_)	mm day^−1^
Transpiration from the Surface Soil (*E*_*Ts*_)	mm day^−1^
Infiltration (*I*)	mm day^−1^
Duration of E-SMAP Interval	days
Scale Factor	NA

*E*_*soil*_ represents the average soil evaporation over valid E-SMAP intervals and is reported for E-SMAP calculations as well as evaluation datasets (GLEAM, Noah and Mosaic) temporally matched to E-SMAP’s screened intervals. Other reported variables represent the average flux over valid E-SMAP intervals as well. All fluxes are reported at the mid-date in E-SMAP intervals.

E-SMAP is compared with one remote sensing-based and two LSM-based soil evaporation datasets in the “Technical Evaluation” (Table 3). The three evaluation datasets were remapped to SMAP’s 9 km EASE-Grid using bilinear interpolation from the CDO software^47^ prior to comparison with E-SMAP. No true ‘validation’ of E-SMAP was conducted because no continental-scale and spatially representative observations of *E*_*soil*_ exist. Thus, the technical evaluation examines similarities and differences of E-SMAP relative to widely used *E*_*soil*_ datasets rather than quantifying the accuracy of E-SMAP. A point scale evaluation of the E-SMAP methodology over 10 validation sites can be found in Small *et al*.^11^.

**Table 3:** Data sources used in evaluating the E-SMAP dataset.

Dataset	Spatial resolution	Temporal resolution	Period of record		Reference and location of data retrieval
**Remote sensing**					
GLEAM	0.25°	daily	1980–2018		Miralles *et al*.^3^ & Martens *et al*.^30^ (https://www.gleam.eu/)
**LSM**					
NLDAS-2 Noah and Mosaic models	0.25°	hourly	1979-Present		Xia *et al*.^21^; Xia *et al*.^22^; NCEP/EMC (2009)^49^ (https://disc.gsfc.nasa.gov/datasets?keywords=NLDAS&page=1)

## Technical Evaluation

**Fig. 3:**
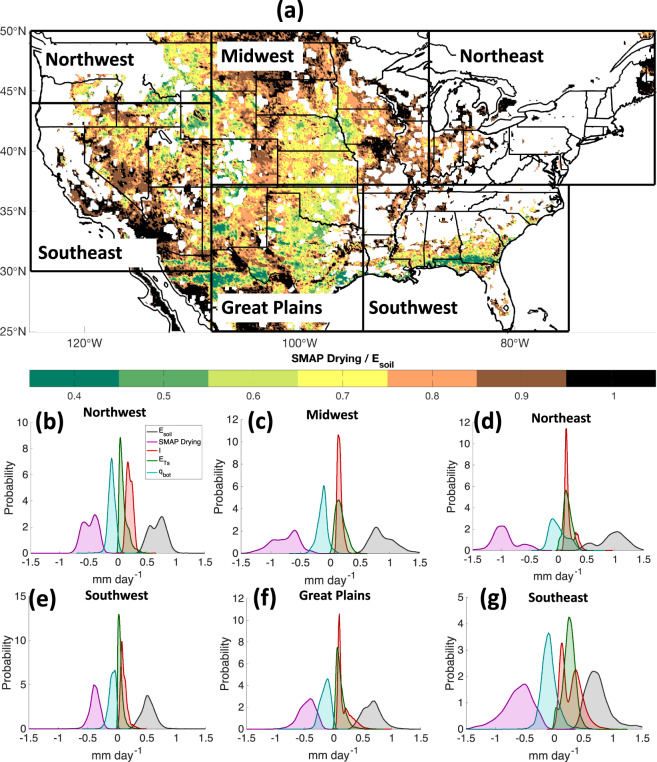
E-SMAP *E*_*soil*_ relies more heavily on observed drying rates than ancillary data and models. **(a)** Median SMAP drying rates divided by *E*_*soil*_ over the E-SMAP domain. Kernel density estimators for each water balance component in Eq. 1 for the **(b)** Northwest, **(c)** Midwest, **(d)** Northeast, **(e)** Southwest, **(f)** Great Plains and **(g)** Southeast. Data presented in panels **a**–**g** are representative of all time steps for each region in the E-SMAP data set.

Kernel density estimators are used to show the overall tendencies of E-SMAP components in Fig. 3b–g. *E*_*soil*_ is largely explained by SMAP drying rates, $-\frac{d{\theta }_{s}}{dt}D$ and is modulated more modestly by other fluxes in Eq. 1 that are estimated from auxiliary data and models (*q*_*bot*_, *I*, and *E*_*Ts*_). On average, for most regions, *q*_*bot*_ is upwards into the surface control volume and largely ‘cancels out’ with *E*_*Ts*_. Additionally, *q*_*bot*_, *I*, and *E*_*Ts*_ are each approximately four to five times smaller than SMAP drying rates. This results in the summation of *q*_*bot*_, *I*, and *E*_*Ts*_ to be, on average, four times smaller than drying rates observed by SMAP (Fig. 3).

The median ratio between SMAP drying rates and *E*_*soil*_ (Fig. 3a) is used to quantify the central tendency of the fraction of the *E*_*soil*_ signal attributable to SMAP drying rates. For example, in the Midwest, this fraction is 0.85, thus the summation of components estimated from ancillary data and tools (*q*_*bot*_, *I*, and *E*_*Ts*_) composes 15% of the *E*_*soil*_ signal. E-SMAP relies on ancillary data and models more heavily where the ratio of SMAP drying to *E*_*soil*_ is substantially less than 1.0. For example, in the Northwest this ratio is approximately 0.77. There is a statistically significant correlation (*p* < 0.01; R^2^ = 0.91) between mean regional drying rates and the ratio of drying rates divided by *E*_*soil*_, supporting the interpretation that where the SMAP drying rates are relatively large, *q*_*bot*_, *I* and *E*_*Ts*_ play smaller roles in the *E*_*soil*_ calculation. Overall, Fig. 3 supports that variability of *E*_*soil*_ in E-SMAP is primarily explained by SMAP drying rates, with contributions from other estimates ranging from 2% (Northeast) to 23% (Northwest).

**Fig. 4:**
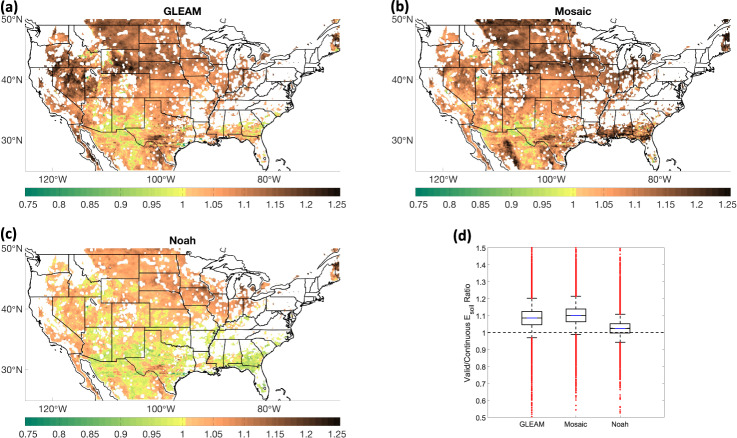
Impact of E-SMAP screening on the magnitude of *E*_*soil*_. The ratio of mean monthly *E*_*soil*_ for valid intervals (after screening) divided by continuous estimates from **(a)** GLEAM, **(b)** Mosaic, and **(c)** Noah. **(d)** Box plot of mean monthly Valid *E*_*soil*_/Continuous *E*_*soil*_, where each whisker is the length of the interquartile range.

We seek to understand the implications of data screening on the magnitude of *E*_*soil*_ to evaluate the representativeness of the screened E-SMAP product on climatological conditions. We compare a screened version of each evaluation product, matching E-SMAP’s temporal sampling produced from screening, with corresponding temporally continuous estimates (Fig. 4). All evaluation datasets show that E-SMAP screening results in a statistically significant increase (*p* < 0.01) in the central tendency of mean monthly *E*_*soil*_ (Fig. 4) and *E*_*soil*_/*ET* (not shown). Evaluation products’ *E*_*soil*_ averaged over valid E-SMAP intervals are larger than corresponding continuous estimates, on average, by 9%, 10% and 2%, while *E*_*soil*_/*ET* is larger by 3%, 17% and 8% for GLEAM, Mosaic and Noah, respectively. Figure 4d shows the interquartile range for the ratio of *E*_*soil*_ from screened time series relative to continuous time series is 1.05–1.12, 1.06–1.14, and 1.00–1.05 for GLEAM, Mosaic and Noah, respectively.

Screening based on negative E-SMAP *E*_*soil*_ results in higher monthly *E*_*soil*_ in all evaluation datasets, whereas precipitation screening results in higher *E*_*soil*_ in GLEAM and Mosaic but lower *E*_*soil*_ from Noah. Precipitation screening results from GLEAM and Mosaic contradict the hypothesis that *E*_*soil*_ is higher over rainy intervals. Therefore, these results may indicate that Noah more accurately represents *E*_*soil*_ relative to GLEAM and Mosaic. However, further analysis into this disagreement is outside the scope of this data descriptor. Regardless, the effect of precipitation screening in reducing Noah *E*_*soil*_ is outweighed by increases corresponding with negativity screening. In sum, all evaluation products show higher *E*_*soil*_ after following the E-SMAP screening procedure. Thus, on average, the E-SMAP product is expected to represent a modest, but significantly higher, monthly *E*_*soil*_ and *E*_*soil*_/*ET* than temporally continuous estimates, notwithstanding large spatial and temporal variability noted in Fig. 4. We therefore include temporally static, gridded scaling factors with the E-SMAP dataset—calculated as the ratio of mean monthly continuous *E*_*soil*_ time series divided by mean monthly screened time series from evaluation datasets—that may be multiplied with E-SMAP’s final *E*_*soil*_ to estimate average temporally continuous *E*_*soil*_ over the 4-year E-SMAP period. Key to the application of these scaling factors is the assumption that *E*_*soil*_ estimated from Eq. 1 is affected by scaling factors similar to evaluation products.

**Fig. 5:**
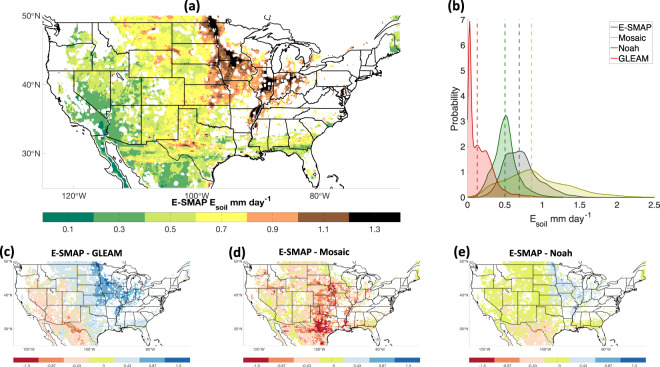
*E*_*soil*_ from E-SMAP is greater than Noah and GLEAM but smaller than Mosaic. **(a)** Mean E-SMAP *E*_*soil*_ over the domain. **(b)** Kernel density estimators of mean *E*_*soil*_ from all locations calculated for E-SMAP, Mosaic, Noah and GLEAM. Vertical dashed lines represent median values. Spatial differences are expressed in mm day^−1^ between E-SMAP and **(c)** GLEAM, **(d)** Mosaic and **(e)** Noah.

*E*_*soil*_ from E-SMAP falls within the range of the evaluation products (Fig. 5). Comparing mean values of *E*_*soil*_, E-SMAP is on average 0.72 mm day^−1^, which is larger than GLEAM (0.17 mm day^−1^) and Noah (0.5 mm day^−1^) but smaller than Mosaic (0.89 mm day^−1^). E-SMAP *E*_*soil*_ has a lower R^2^ with GLEAM, Mosaic and Noah (0.16, 0.13 and 0.15, respectively; not shown) than correlations between the GLEAM and the LSM evaluation datasets (R^2^ = 0.48 and 0.52 with Mosaic and Noah, respectively), which may be reflective of E-SMAP’s independence from these datasets. Reduced correlations are also partially attributable to the SMAP drying rates themselves, which are expected to be unbiased but contain random noise that may exceed the magnitude of *E*_*soil*_ in some cases^32^. This noisiness would correspond with a noisy *E*_*soil*_ estimate with reduced correlation relative to evaluation datasets, but with more stable averages over seasonal or longer time periods. Overall, *E*_*soil*_ from E-SMAP is comparable with *E*_*soil*_ from the evaluation datasets but caution should be exercised with individual data points because the effect of random noise within SMAP drying rates.

## Usage Notes

Moisture flux estimates in the E-SMAP dataset represent the average flux over the valid SMAP interval and are reported at the mid-date of respective intervals. The E-SMAP dataset may be used to estimate soil evaporation over a time period of months or years. However, soil evaporation estimates at individual time steps should be used with caution because unbiased uncertainty in observed drying rates from the SMAP satellite will introduce noise into shorter-interval estimates.

## Data Availability

All scripts are accessible here: https://github.com/RAbolafiaRosenzweig/ESMAP. R code was used for the calculations of each component in Eq. 1 and gridding outputs from individual pixels to the E-SMAP grid. MATLAB was used to produce the final data product and conduct the technical validation. Further, processing of the data in network Common Data Form (netCDF) format was done for remapping and aggregating using the open source Climate Data Operators (CDO) and netCDF Operator (NCO) utilities. Hydrus-1D simulations were performed with publicly available model code (https://github.com/bilke/hydrus).
